# Supplementary material for: Predictors of Seasonal Influenza Vaccination Willingness among High-Risk Populations Three Years after the Onset of the COVID-19 Pandemic
Source: Vaccines (Basel). 2023 Feb 1;11(2):331. doi: 10.3390/vaccines11020331 (PMC9963446; doi:10.3390/vaccines11020331)
Supplement: Supplementary file 1 [file vaccines-11-00331-s001.zip › vaccines-2191323-supplementary.pdf]

Supplementary Table S1. Sensitivity analysis after the removal of the variable "COVID-19 booster doses" from the univariate and multivariable linear models (dependent variable: influenza vaccination willingness score).

**Independent variables**	**Univariate model**		**Multivariable model**	
	**Unadjusted coefficient beta (95% CI)**	**P-value**	**Adjusted coefficient beta (95% CI)[a]**	**P-value**
Gender (females vs. males)	1.204 (-2.333 to -0.075)	0.037	-0.396 (-1.531 to 0.739)	0.493
Age (years)	0.068 (0.035 to 0.101)	<0.001	0.045 (0.008 to 0.083)	**0.018**
Educational level (university degree vs. high school)	0.420 (-0.703 to 1.543)	0.462	0.526 (-0.592 to 1.644)	0.355
Self-perceived health status (good/very good vs. very poor/poor/moderate)	-0.419 (-1.572 to 0.735)	0.476	-0.106 (-1.282 to 1.069)	0.859
SARS-CoV-2 infection (yes vs. no)	0.138 (-0.836 to 1.112)	0.780	0.387 (-0.542 to 1.316)	0.387
Adverse effects because of COVID-19 vaccination	-0.361 (-0.535 to -0.187)	<0.001	-0.235 (-0.432 to -0.038)	**0.019**
Resilience	-0.003 (-0.634 to 0.628)	0.992	-0.302 (-1.062 to 0.457)	0.434
Family support	0.360 (0.034 to 0.686)	0.031	0.687 (0.223 to 1.152)	**0.004**
Friends support	-0.206 (-0.512 to 0.101)	0.187	-0.298 (-0.706 to 0.109)	0.150
Significant others support	-0.123 (-0.457 to 0.211)	0.467	-0.307 (-0.830 to 0.216)	0.249
Anxiety	-0.027 (-0.275 to 0.220)	0.828	0.332 (-0.056 to 0.719)	0.093
Depression	-0.070 (-0.329 to 0.188)	0.593	-0.187 (-0.609 to 0.236)	0.384
COVID-19-related emotional exhaustion	-0.195 (-0.579 to 0.189)	0.318	-0.039 (-0.603 to 0.525)	0.892
COVID-19-related physical exhaustion	0.053 (-0.349 to 0.455)	0.794	0.384 (-0.234 to 1.001)	0.224
Exhaustion due to measures against the COVID-19	-0.512 (-0.866 to -0.159)	0.005	-0.581 (-1.009 to -0.153)	**0.008**

Bold p-values indicate statistically significant associations in the multivariable model. CI: confidence interval

[a] p-value for ANOVA<0.001; R^2 for the final multivariable model was 12.8%
